# Genome-wide expression profiling shows transcriptional reprogramming in *Fusarium graminearum* by Fusarium graminearum virus 1-DK21 infection

**DOI:** 10.1186/1471-2164-13-173

**Published:** 2012-05-06

**Authors:** Won Kyong Cho, Jisuk Yu, Kyung-Mi Lee, Moonil Son, Kyunghun Min, Yin-Won Lee, Kook-Hyung Kim

**Affiliations:** 1 Department of Agricultural Biotechnology, Center for Fungal Pathogenesis and Research Institute for Agriculture and Life Sciences, Seoul National University, Seoul, 151-921, Republic of Korea

## Abstract

**Background:**

Fusarium graminearum virus 1 strain-DK21 (FgV1-DK21) is a mycovirus that confers hypovirulence to *F. graminearum*, which is the primary phytopathogenic fungus that causes Fusarium head blight (FHB) disease in many cereals. Understanding the interaction between mycoviruses and plant pathogenic fungi is necessary for preventing damage caused by *F. graminearum*. Therefore, we investigated important cellular regulatory processes in a host containing FgV1-DK21 as compared to an uninfected parent using a transcriptional approach.

**Results:**

Using a 3′-tiling microarray covering all known *F. graminearum* genes, we carried out genome-wide expression analyses of *F. graminearum* at two different time points. At the early point of growth of an infected strain as compared to an uninfected strain, genes associated with protein synthesis, including ribosome assembly, nucleolus, and ribosomal RNA processing, were significantly up-regulated. In addition, genes required for transcription and signal transduction, including fungal-specific transcription factors and cAMP signaling, respectively, were actively up-regulated. In contrast, genes involved in various metabolic pathways, particularly in producing carboxylic acids, aromatic amino acids, nitrogen compounds, and polyamines, showed dramatic down-regulation at the early time point. Moreover, genes associated with transport systems localizing to transmembranes were down-regulated at both time points.

**Conclusion:**

This is the first report of global change in the prominent cellular pathways in the *Fusarium* host containing FgV1-DK21. The significant increase in transcripts for transcription and translation machinery in fungal host cells seems to be related to virus replication. In addition, significant down-regulation of genes required for metabolism and transporting systems in a fungal host containing the virus appears to be related to the host defense mechanism and fungal virulence. Taken together, our data aid in the understanding of how FgV1-DK21 regulates the transcriptional reprogramming of *F. graminearum*.

## Background

*Fusarium graminearum* (teleomorph *Gibberella zeae*) is a well known phytopathogenic fungus associated with Fusarium head blight (FHB) disease, which causes blights, root rots, or wilts, especially in economically important cereal crops such as wheat, maize, and barley [1]. FHB is considered an important fungal disease because it drastically reduces grain yield and quality, and produces mycotoxins such as deoxynivalenol (DON) and nivalenol (NIV) in cereals, which are very harmful to human and animal health [2,3]. The fungus can also infect several dicotyledonous plants including *Arabidopsis*, tobacco, tomato, and soybean [4].

Viruses that infect plant fungi are referred to as mycoviruses. Infection by some mycoviruses confers hypovirulence by attenuating pathogenicity to their fungal hosts, which are mostly plant pathogens. Mycoviruses tend to be double-stranded RNA (dsRNA) viruses [5], and several *Fusarium*-infecting mycoviruses have been isolated [6-8]. In addition, several whole genome sequences of dsRNA mycoviruses strains derived from *F. graminearum* have recently been reported [9-12].

In many cases, such as those of *F. poae* (*Fusarium poae virus 1*, FpV1) [7] and *F. solani* (*Fusarium solani virus 1*, FsV1), viral infection is not associated with phenotypic changes [8]. However, Fusarium graminearum virus 1 strain-DK21 (FgV1-DK21) exhibits interesting phenotypes including reduced mycelial growth and the induction of dark red pigmentation [6]. Several previous studies have provided strong evidence that hypovirulent mycoviruses could be used as substitutes for fungicides [13,14]. A recent study demonstrated that protoplast fusion is the most efficient approach for transmitting mycoviruses among a wide range of phytopathogenic fungi and that this approach will facilitate the use of mycoviruses as a biocontrol agent [15].

With the increasing availability of whole genome sequences for representative plant fungal pathogens [16], extensive and diverse genome-wide analyses can be performed, including transcriptomics, proteomics, and metabolomics [17]. Proteomics approaches for different *Fusarium* species have enabled examinations of extracellular proteins, proteins involved in fumonisin biosynthesis, and proteome profiles upon antagonistic rhizobacteria inoculation and mycovirus infection [18-21]. Several gene expression analyses based on microarrays have also been conducted [21-25]. For example, genome-wide expression profiling of *F. graminearum* was carried out to examine responses to treatment with azole fungicide tebuconazole and during perithecium development [22,24]. Microarrays provide a valuable tool for detecting and identifying *Fusarium* species that produce specific metabolites such as trichothecene and moniliformin [23,25]. Moreover, the recently completed genome sequencing of three major *Fusarium* species provides an important resource for studying pathogenicity and functions of individual genes [26].

Several microarray-based studies have demonstrated transcriptional changes in fungal genes following mycovirus infection, although most of these studies examined only CHV1-713 infecting the chestnut blight fungus *Cryphonectria parasitica*. Initially, a polymerase chain reaction (PCR)-based approach demonstrated that elevation of cAMP levels by CHV1-713 resulted in reduced accumulation of the GTP-binding (G) protein subunit CPG-1 [25]. In addition, cDNA microarrays containing 2,200 genes from *C. parasitica* showed transcriptional change in G-signaling pathways following hypovirus infections showing different virulence or phenotypes [27-29].

Infection by a virus leads to changes in diverse biological processes between fungal host and viral factors. It is of interest to examine such alterations at the molecular level. However, no previous reports have examined expression differences between a fungus containing a mycovirus and an infected parent, aside from two papers that used microarray cDNA chips based on expressed sequence tags to examine fungal host gene expression upon mycovirus infection [28,29]. Here, we examined genome-wide transcriptional differences in *F. graminearum* expression between a strain harboring FgV1-DK21 and its uninfected parent. This is the first report of a genome-wide fungal gene expression analysis during mycovirus infection using a 3′ tiling microarray, and our findings show global differences in host cellular pathways in *F. graminearum* harboring FgV1-DK21.

## Results

### Genome-wide 3′-tiling microarray to identify differentially expressed genes in *F. graminearum* harboring FgV1-DK21

**Figure 1:**
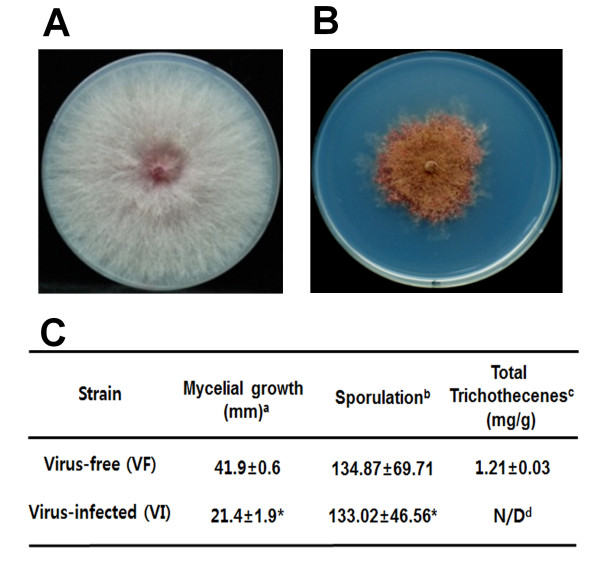
**Characteristics of virus-free *F. graminearum* and FgV1-DK21-infected *F. graminearum *.** (**A**) Virus-free *F. graminearum* (**B**) FgV1-DK21-infected *F. graminearum*. (**C**) Morphological differences between virus-free and virus-infected *F. graminearum*. ^a^Radial growth was measured on PDA 120 h after inoculation. ^b^The number of spores was measured in CMC broth at 25°C for 9 days. The number of spores indicated represents the number per 1 ml. Data were described previously [6]. ^c^Production of deoxynivalenol and 15-acetyldeoxynivalenol. For toxin analysis, all strains were grown in minimal medium supplemented with 5 mM agmatine and incubated for 7 days. ^d^N/D: not detected. ^*^ The value is significantly different than that of the virus-free isolate as determined by a Student’s *t*-test.

**Figure 2:**
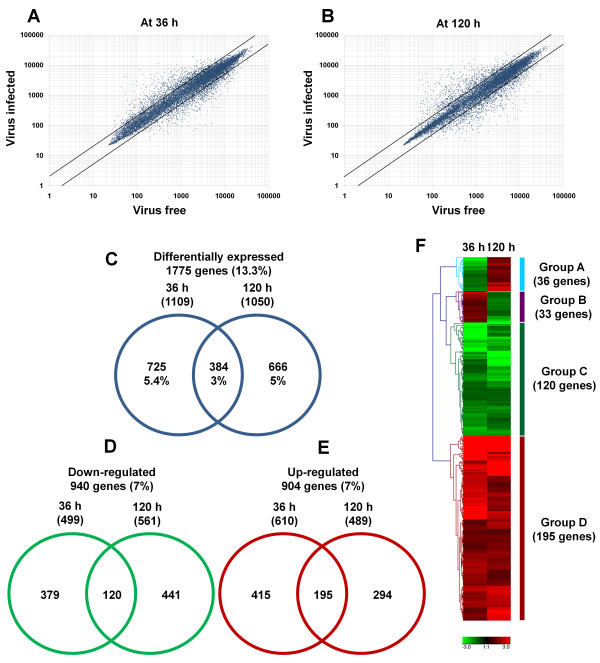
**Differentially expressed *F. graminearum* genes during FgV1-DK21 infection identified by microarray.** Scatterplots of normalized signal intensities 36 h (**A**) and 120 h (**B**) after inoculation with FgV1-DK21 as compared to virus-free samples. All signal intensities were converted to a log10 scale. The diagonal lines indicate a two-fold change. The Venn diagram illustrates the total number of genes that were significantly differentially expressed at 36 h and 120 h (**C**), down-regulated genes (**D**), and up-regulated genes (**E**). The heat map shows the expression patterns of 384 genes that were hierarchically clustered and differentially expressed at both 36 and 120 h (**F**). Red and green indicate up-regulation and down-regulation, respectively.

The virus-infected *F. graminearum* exhibited strong inhibition of mycelia growth as well as reduced levels of DON at 7 days after inoculation (Figure 1). To visualize how gene expression patterns were affected at different time points, we generated scatterplots (Figure 2). Interestingly, the scatterplots showed that there were no significant differences in the number of differentially expressed genes between 36 h and 120 h; however, it appeared that the changes in gene expression at 120 h were somewhat more extensive than those at 36 h (Figure 2). To identify differentially expressed genes, we first performed hierarchical clustering, which identified gene sets of significantly differentially expressed genes at two different time points (Additional file 1: Table S1 and Additional file 2: Table S2). Most of the identified genes showed at least two-fold differential expression. A total of 1775 genes, representing 13.3% of 13,382 genes, were differentially expressed at both time points (Figure 2C), with 1109 (5.4%) and 1050 genes (5%) identified as differentially expressed at 36 h and 120 h, respectively (Figure 2C). Moreover, 384 genes (3%) were differentially expressed at both time points (Figure 2C).

We further analyzed the lists of differentially expressed genes according to those down-regulated vs. those up-regulated (Figure 2D, E). Genes with an adjusted P-value less than 0.05 were selected as differentially expressed genes. In both time points, 940 genes (7%) were down-regulated whereas 904 genes (7%) were up-regulated (Figure 2D, E). Among the down-regulated genes, there were more differentially expressed genes at 120 h (561 genes) than at 36 h (499 genes) (Figure 2D). In contrast, more genes were induced at 36 h (610 genes) than at 120 h (489 genes) (Figure 2E). Moreover, 120 genes and 195 genes were commonly found in the group of down-regulated and up-regulated genes, respectively (Figure 2D, E). We then investigated the expression patterns of 384 genes that were differentially expressed at both time points using hierarchical clustering, which sorted the genes into four groups according to expression patterns (Figure 2F). Group A contained 36 genes that were highly down-regulated at 36 h but were up-regulated at 120 h. In contrast, the 33 genes belonging to group B were highly induced at 36 h, and subsequently down-regulated (Figure 2F). Group C included 120 genes that were strongly repressed regardless of virus infection time, and group D contained 195 genes that showed consistently elevated gene expression across both time points.

### Representative genes showing significant expression change and real-time validation of the microarray data by quantitative real-time reverse transcription PCR

Representative fungal genes that showed significant gene expression are listed in Table 1. At 36 h, several genes encoding enzymes including phospholipase/carboxylesterase, polyketide synthase, eukaryotic aspartyl protease and dipeptidyl aminopeptidases were highly induced, whereas transcripts involved in transport, such as amino acid transporter permease and ABC transporter, were up-regulated at 120 h (Table 1). In contrast, several of the repressed genes at 36 h included maltose transporter, linoleate diol synthase, and genes with unknown functions, whereas those encoding ferric reductase and abhydrolase 3 were strongly repressed at 120 h (Table 1).

**Table 1:** Representative *F. graminearum* genes exhibiting significant differential expression levels in microarray analysis

**Gene locus**	**Function**	**Log**_**2**_**FC**	**P-value**	**Time point**
**Ten representative up-regulated genes**				
FGSG_07801	Phospholipase/Carboxylesterase	6.487887158	0.001776791	36 h
FGSG_07798	Polyketide synthase	6.350204512	0.001744378	36 h
FGSG_07800	Eukaryotic aspartyl protease	6.046050259	0.004179133	36 h
FGSG_13222	Dipeptidyl aminopeptidases	5.960147173	0.003763144	36 h
FGSG_07804	Cytochrome P450	5.427966104	0.011883416	36 h
FGSG_04468	Amino acid transporter permease	4.672200299	0.012719315	120 h
FGSG_08055	Amino acid transporter permease	4.288189196	0.013683756	120 h
FGSG_03788	Glyco_hydrolase_16	4.044638062	0.013683756	120 h
FGSG_05076	ABC transporter	3.965527131	0.012719315	120 h
FGSG_03687	Esterase lipase	3.836457968	0.012719315	120 h
**Ten representative down-regulated genes**				
FGSG_03911	Maltose transporter	−5.371438178	0.046804957	36 h
FGSG_02668	Linoleate diol synthase	−5.234881513	0.014192107	36 h
FGSG_10572	unknown function	−4.787897398	0.028984152	36 h
FGSG_11146	Linoleate diol synthase	−4.688195689	0.027402009	36 h
FGSG_04795	unknown function (DUF1612)	−4.567155895	0.013485652	36 h
FGSG_03143	Glycosyl hydrolase 88	−5.893401797	0.018275961	120 h
FGSG_03737	Major facilitator	−5.229057946	0.012719315	120 h
FGSG_04780	Ferric reductase	−4.889869656	0.03402279	120 h
FGSG_11119	Ferric reductase	−4.796036501	0.015887627	120 h
FGSG_03738	Abhydrolase 3	−4.655190203	0.015033887	120 h

**Figure 3:**
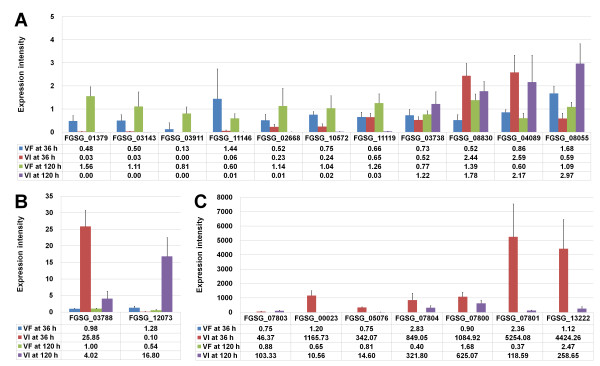
**Validation of microarray data by qRT-PCR.** To validate the results of the microarray data, we selected 20 *F. graminearum* genes showing strong expression ratios by microarray analysis. Due to the different expression intensities among genes, we divided the 20 genes into three groups for better visualization of qRT-PCR results: expression intensities less than 4 (**A**), between 4 and 100 (**B**), and more than 100 (**C**). Abbreviations: VF, virus-free; VI, virus-infected. The number in the table indicates the expression intensity of each gene.

To validate the microarray data, we selected 20 genes whose expression was significantly affected in the host containing FgV1-DK21, as demonstrated by the microarray analysis, and determined their relative expression by quantitative real-time reverse transcription PCR (qRT-PCR) (Figure 3 and Additional file 3: Table S3 and Additional file 4: Table S4). Genes that showed diverse expression patterns were categorized into different functional classes (Figure 3 and Additional file 3: Table S3). The results of the qRT-PCR were highly consistent with those of the microarray data. For example, according to the qRT-PCR and microarray results, the transcript levels for three genes, including FGSG_01379, FGSG_03143, and FGSG_03911, were highly reduced at both 36 h and 120 h, whereas FGSG_03788, FGSG_00023, FGSG_07804, FGSG_07801, and FGSG_13222 were strongly induced regardless of the time point (Figure 3A–C). When the mRNA level of a gene is too low to quantify, or the P-values from the microarray data are very high, it is highly likely that qRT-PCR results will not correlate with microarray data, as was observed for FGSG_11119 and FGSG_04089 (Figure 3A). As compared to the microarray approach, qRT-PCR offers a highly sensitive technique for detecting low amounts of transcripts and provides the transcript level for the gene of interest. For example, the expression intensities for 11 genes were relatively low, ranging from 0 to 2.97 (Figure 3A), whereas the amount of mRNAs for NAD-dependent aldehyde dehydrogenases (FGSG_07801) and dipeptidyl aminopeptidases (FGSG_13222) were very high and ranged from 4424.26 to 5254.08, particularly in the host containing FgV1-DK21 at 36 h (Figure 3C).

### FunCat classification for an overview of the transcriptional reprogramming of *F. graminearum* harboring FgV1-DK21

We subjected a total of 1775 differentially expressed genes to functional catalogue (FunCat) annotation to gain insight into their functional classifications [29]. Specifically, we divided differentially expressed genes into four groups (Table 2). More than half of the differentially expressed genes were not assigned to any functional category. Specifically, 344 genes (71.9%) in the group of down-regulated genes at 120 h were unclassified. Of the functional categories, the vast majority of differentially expressed genes were associated with metabolism (Table 2). Note that 118 genes (28.2%) were down-regulated while 81 genes (22.9%) were up-regulated at 36 h, whereas there were more up-regulated genes (95) than down-regulated genes (79) at 120 h. Based on the number of differentially expressed genes, it is likely that genes involved in various metabolic pathways were severely repressed at 36 h and then were gradually induced at 120 h. Along with a gene set for metabolism, genes associated with energy were highly down-regulated (26 genes) at 36 h (Table 2). In contrast, genes involved in transcriptional and translational machinery were dominantly up-regulated early after FgV1-DK21 infection. For example, genes associated with the cell cycle and DNA processing (21 genes), transcription (46 genes), protein synthesis (35 genes), protein fate (18 genes), and those encoding proteins with binding function (81 genes) were highly up-regulated at 36 h. The number of down-regulated genes associated with cellular transport at 36 h was almost twice that of up-regulated genes. Conversely, the number of up-regulated genes that govern cellular transport was similar to that of the down-regulated genes at 120 h.

**Table 2:** Gene functions of significantly differentially expressed *F. graminearum* genes based on annotation of functional categories in FunCat

**Functional category**	**Down-regulation at 36 h**	**Up-regulation at 36 h**	**Down-regulation at 120 h**	**Up-regulation at 120 h**	**Whole genome**
Metabolism	118 (28.2%)	81 (22.9%)	79 (16.5%)	95 (22.9%)	2324 (16.9%)
Energy	26 (6.22%)	12 (3.39%)	13 (2.71%)	15 (3.62%)	503 (3.66%)
Cell cycle and DNA processing	7 (1.67%)	21 (5.94%)	2 (0.41%)	10 (2.41%)	660 (4.81%)
Transcription	4 (0.95%)	46 (13.0%)	1 (0.2%)	7 (1.69%)	720 (5.24%)
Protein synthesis	N.D	35 (9.91%)	1 (0.2%)	2 (0.48%)	370 (2.69%)
Protein fate	9 (2.15%)	18 (5.09%)	9 (1.88%)	13 (3.14%)	920 (6.70%)
Protein with binding function	40 (9.56%)	81 (22.9%)	24 (5.02%)	49 (11.8%)	1716 (12.5%)
Regulation of metabolism	N.D	3 (0.84%)	3 (0.62%)	1 (0.24%)	242 (1.76%)
Cellular transport	75 (17.9%)	38 (10.7%)	51 (10.6%)	52 (12.5%)	1391 (10.1%)
Cellular communication	5 (1.19%)	11 (3.11%)	3 (0.62%)	4 (0.96%)	312 (2.27%)
Cell rescue, defense, and virulence	28 (6.69%)	38 (10.7%)	29 (6.06%)	45 (10.8%)	859 (6.26%)
Interaction with the environment	20 (4.78%)	21 (5.94%)	23 (4.81%)	26 (6.28%)	607 (4.42%)
Systemic interaction with the environment	N.D	1 (0.28%)	N.D	N.D	12 (0.08%)
Cell fate	2 (0.47%)	6 (1.69%)	4 (0.83%)	2 (0.48%)	240 (1.74%)
Development	4 (0.95%)	1 (0.28%)	2 (0.41%)	1 (0.24%)	55 (0.40%)
Biogenesis of cellular components	13 (3.11%)	18 (5.09%)	10 (2.09%)	13 (3.14%)	617 (4.49%)
Cell type differentiation	1 (0.23%)	11 (3.11%)	3 (0.62%)	6 (1.44%)	273 (1.98%)
Unclassified	241 (57.6%)	333 (54.59%)	344 (71.9%)	276 (66.6%)	8894 (64.8%)

N.D. indicates that genes were not detected in a given functional category.

Gray indicates the number of genes that were significantly enriched.

### FgV1-DK21 changes the transcript levels of fungal genes involved in ribosome biogenesis

**Figure 4:**
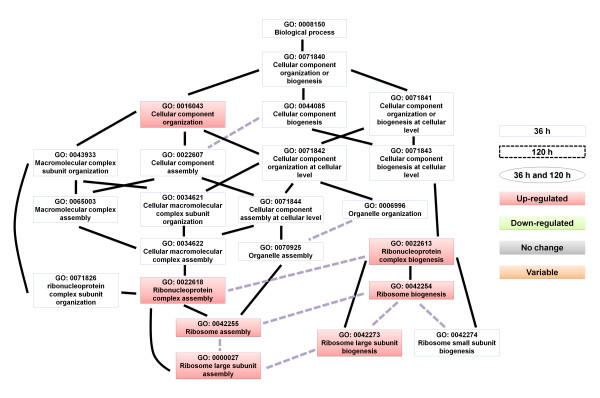
**A GO diagram of significantly over-represented GO terms related to biological processes, particularly highlighting cellular components.** Significantly enriched GO terms are illustrated by directed acylic graphs (DAG). The closed rectangles, dotted-rectangles, and circles indicate enriched GO terms for 36 h, 120 h, and both time points, respectively. Black and gray dotted lines represent direct and partial interactions with higher GO terms, respectively. Rectangles and circles filled with the respective color indicate enriched GO terms. Red, green, gray, and orange colors indicate up, down, no change, and variable expression changes, respectively.

**Figure 5:**
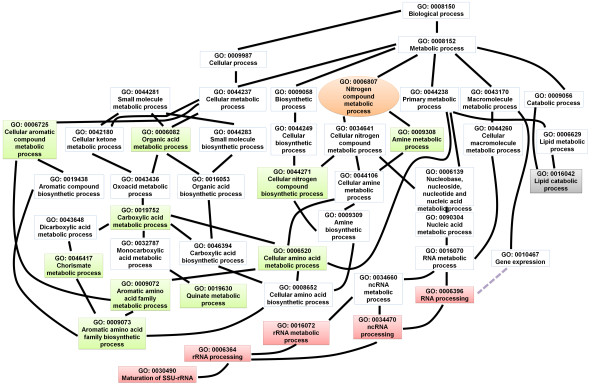
GO diagram of significantly over-represented GO terms in biological processes associated with metabolism.

**Figure 6:**
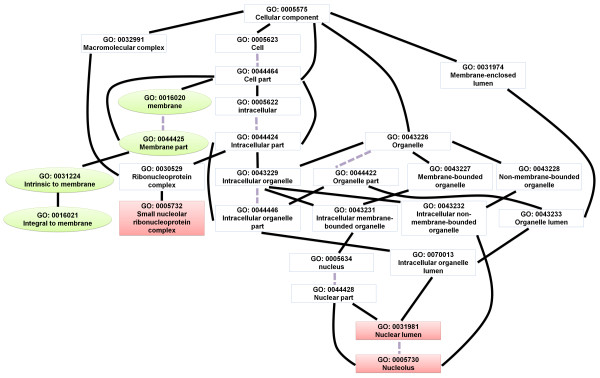
GO diagram of significantly over-represented GO terms in cellular components.

**Figure 7:**
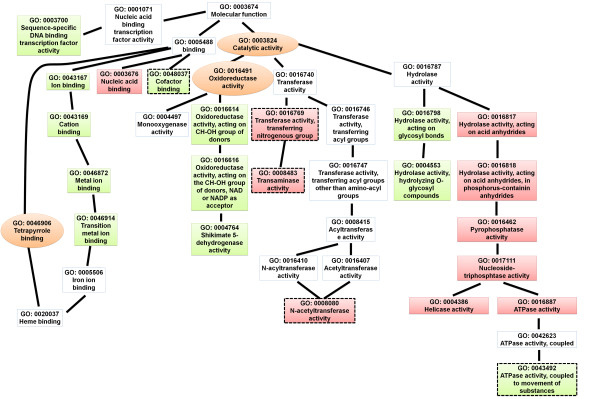
GO diagram of significantly over-represented GO terms in molecular function, particularly those associated with catalytic activity.

**Figure 8:**
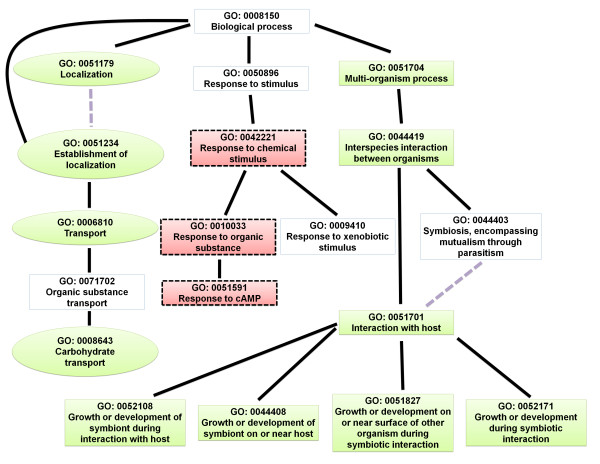
GO diagram of significantly over-represented GO terms in biological processes associated with transport, response to stimulus, and interaction with host.

**Figure 9:**
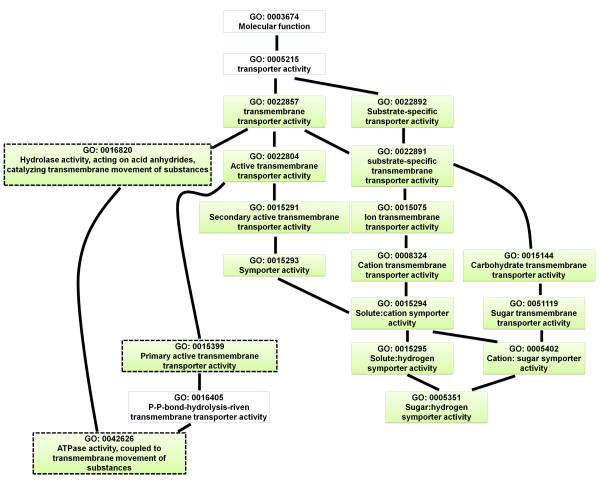
GO diagram of significantly over-represented GO terms in molecular function, particularly those associated with transporter activity.

Next, we analyzed the enriched gene ontology (GO) terms of the differentially expressed genes. The identified enriched GO terms are listed in Additional file 5: Table S5. The directed acyclic graph (DAG) illustrates the GO terms that were over-represented (Figures 4, 5, 6, 7, 8, and 9). Interestingly, GO terms related to ribosome biogenesis, such as the ribosome ribonucleoprotein complex assembly (GO: 0022618), ribosome assembly (GO: 0042255), ribonucleoprotein complex biogenesis (GO: 0022613), and ribosome biogenesis (GO: 0042254), were highly over-represented (Figure 4). Moreover, GO terms for RNA processing (GO: 0006396), ncRNA processing (GO: 0034470), rRNA metabolic processes (GO: 0016072), rRNA processing (GO: 0006364), and maturation of SSU-rRNA (GO: 0030490) were over-represented with high levels of transcripts (Figure 5). Similarly, GO terms related to the nucleolus, such as the small nucleolar ribonucleoprotein complex (GO: 0005732), the nuclear lumen (GO: 0031981), and nucleolus (GO: 0005730), were over-represented (Figure 6). Transcripts associated with the nucleolus were highly accumulated at 36 h.

### Influence of FgV1-DK21 on fungal host gene expression related to metabolic pathways and stress responses

It was not surprising that the expressions of genes that involve a range of metabolic pathways were significantly affected by FgV1-DK21 infection. Consistent with the FunCat annotation, GO enrichment analysis showed that genes associated with a variety of metabolic pathways were significantly down-regulated, particularly at 36 h. For example, over-represented GO terms included cellular aromatic compound metabolic processes (GO: 0006725), organic acid metabolic processes (GO: 0006082), oxoacid metabolic processes (GO: 0043436), carboxylic acid metabolic processes (GO: 0019752), chorismate metabolic processes (GO: 0046417), aromatic amino acid family biosynthetic processes (GO: 0009073), quinate metabolic processes (GO: 0019630), amine metabolic processes (GO: 0009308), and cellular nitrogen compound biosynthetic processes (GO: 0044271) (Figure 5). Most genes related to over-represented metabolic pathways tended to be down-regulated at 36 h. In contrast, the molecular functions for those genes were consistent with the highly up-regulated GO terms for transferase activity, transferring nitrogenous group (GO: 0016769), transaminase activity (GO: 0008483), and N-acetyltransferase activity (GO: 0008080) at 120 h (Figure 7).

In particular, genes involved in several stress responses, such as to chemical stimulus (GO: 0042221), organic substances (GO: 0010033), and cAMP (GO: 0051591), were up-regulated, especially at 120 h (Figure 8). Of the identified GO terms, those reflecting interactions with the fungal host (GO: 0051701) as well as growth or development of symbionts during interactions with the fungal host (GO: 0052108) at 36 h are expected results (Figure 8). All genes associated with host interactions showed highly down-regulated gene expression.

### Down-regulation of membrane-associated transporter genes

In the cellular component ontology, GO terms associated with membranes (GO terms 0016020, 0044425, 0031224, and 0016021) were over-represented (Figure 6). Interestingly, genes associated with membranes were down-regulated regardless of the time point (Figure 6). Furthermore, transporter activity was one of the significantly over-represented GO terms with respect to molecular function. Of a total of 17 GO terms for transporter activity, 14 were present at 36 h, including carbohydrate transmembrane transporter activity (GO: 0015144), ion transmembrane transporter activity (GO: 0015075), and sugar:hydrogen symporter activity (GO: 0005351) (Figure 9). At 120 h, three GO terms, including primary active transmembrane transporter activity (GO: 0015399), ATPase activity, and coupled to transmembrane movement of substances (GO: 0042626), were over-represented (Figure 9).

### *F. graminearum* transcription factors participate in transcription regulation of the host containing FgV1-DK21

Transcription factors (TFs) play a key role in signal transduction pathways by regulating gene expression to control biological processes [30]. Thus, it is of interest to understand their involvement in fungi–virus interactions at the transcriptional level. Whole genome sequences of *F. graminearum* show that there are at least 659 TFs divided into 44 families [31]. We identified more differentially expressed TFs at the early time point (57 TFs) than at the late time point (27 TFs) ( Additional file 6: Table S6). Zn2Cys6 (42 TFs) was the most prevalent TF family, followed by the C2H2 zinc finger (7 TFs) and bHLH (2 TFs) families. In addition, centromere protein B, DNA-binding region, homeodomain-like, Lambda repressor-like, DNA-binding, nucleic acid-binding, OB-fold, TF jumonji, and bZIP were also identified as TFs showing significant change at the transcript level ( Additional file 6: Table S6). Zn2Cys6 (15 TFs) was also the most prominent TF family at the late time point, followed by C2H2 zinc finger (3 TFs), bHLH (2 TFs), and bZIP (2 TFs) ( Additional file 6: Table S6). Moreover, the gene expressions of TFs belonging to the Myb (a negative transcriptional regulator), TF jumonji, zinc finger (CCHC-type), and zinc finger (NF-X1-type) families were significantly changed. Recently, a mutant library of 657 putative TFs was established via homologous recombination in *F. graminearum*, providing a valuable resource to study gene regulation in fungus [32]. Thus, it might be of interest to compare phenotypes of mutants for the 75 differentially expressed TFs. Most TF knock-out mutants show distinct phenotypes. For example, five mutants for which members of the C2H2 zinc finger family were deleted (*FGSG_04083* and *FGSG_12837*) and Zn2Cys6 (*FGSG_04901*, *FGSG_05789*, and *FGSG_09464*) displayed abnormal phenotypes compared to wild type controls. Interestingly, the deletion of *FGSG_04083* resulted in a hypervirulence phenotype. The expression of all five of these TFs was strongly up-regulated 36 h after virus infection. Two genes (*FGSG_12837* and *FGSG_09464*) were highly expressed in both early and late time points. The ascospore was not formed in *FGSG_12837* mutants, while that for *FGSG_09464* exhibited increased resistance to oxidative stress.

## Discussion

### FgV1-DK21 drastically induces the expression of fungal host genes required for their replication

Perhaps the most striking finding of this study is that the host containing FgV1-DK21 accumulates transcripts associated with translation machinery, such as ribosome biogenesis and the nucleolus. Ribosomes function in the production of proteins, while the nucleolus is the site of ribosomal RNA synthesis and ribosome assembly [33]. The nucleolus actively participates in a variety of biological processes, including cell cycle regulation, cell growth, stress sensing, and viral infection [34]. Moreover, genes involved in RNA processing were highly induced. These genes convert precursor RNAs such as non-coding RNA (ncRNA) and small subunit (SSU) ribosomal RNAs molecules into mature RNA molecules. Taken together, these findings suggest that the entire complex for protein synthesis and processing in fungal host cells was highly activated by FgV1-DK21.

Viruses rely on host cell machinery and have evolved sophisticated mechanisms to achieve replication efficiently during virus infection [35]. Given that nucleolus localizing genes were up-regulated, it appears that the virus stimulates gene expression associated with nucleolus. As a result, the nucleolus produces numerous ribosomes to maximize viral replication. In addition, the virus might control protein synthetic machinery in host ribosomes to replicate viral proteins. Recently, proteomics-based studies confirmed the involvement of the nucleolus in viral infection and replication [36,37]. Previous studies have shown that RNA viruses can interact with several nucleolar proteins such as nucleolin, B23, and fibrillarin to facilitate virus replication [38]. Thus, it might be of interest to examine the interactions between nucleolar proteins from *F. graminearum* and FgV1-DK21 viral proteins in future studies. Although our data suggest that expression of genes related to ribosomes was strongly affected by virus infection, this might be a common phenomenon in hosts in response to various kinds of virus. Thus, we cannot exclude the possibility that ribosomes are indirectly involved in the replication of dsRNA viruses. Since little is known about dsRNA viruses, we refer to many results from single-stranded (ss) RNA viruses to support our data. The system for dsRNA viruses might differ from that of ssRNA viruses.

### Genes associated with metabolic pathways play a critical role in the fungal defense mechanism against viral infection

Metabolism is the core of cellular functions, and comprises numerous reactions that function in the degradation of nutrients and biosynthesis of cellular components including proteins, lipids, carbohydrates, DNA, and RNA. Our results, as well as a previous report of *C. parasitica*, found dramatic differences in gene expression associated with metabolic pathways [39]. However, there are inherent differences between that study and the present one. Specifically, our microarray data demonstrated down-regulation of genes involved in metabolism. In contrast, the previous study found that the majority of metabolites, including lipids and carbohydrates, were significantly accumulated [39]. This difference may be due to different infection times. We found the most dramatic changes in gene expression at the early time point, whereas metabolic probing showed that a variety of metabolites accumulated with increasing infection time [39].

Differential expression of genes related to metabolism might be associated with the altered host phenotype. For example, in the group of down-regulated genes at 36 h, genes for cell type differentiation were also highly enriched, suggesting that host cell differentiation seems to be induced by viral infection. Interestingly, several studies provide evidence that filamentous differentiation in fungi is required for virulence [40]. Regardless of viral infection stage, genes related to cell rescue, defense, and virulence were highly up-regulated, suggesting that the host defense system was consistently activated. Compared to the whole genome, genes with significantly enriched functions were mostly found in the group showing up-regulation at 36 h, suggesting that the transcriptional regulation in the host harboring mycovirus is more important at the early time point than the late time point.

Of the altered metabolites, our study as well as a previous report [39] found dramatic changes in gene expression levels for polyamine production. Polyamines play roles in many biological processes, such as cell growth, development, and responses to various stresses [41]. Thus, it is likely that the down-regulation of genes involved in polyamine biosynthesis during the early stage (36 h) could be correlated with reduced levels of DON, which confers hypovirulence to host *F. graminearum* (Figure 1). A previous study showed that polyamine biosynthesis inhibitors decreased mycelial growth of *Sclerotinia sclerotiorum*[42]. This result is highly consistent with observed phenotypes in virus-infected *F. graminearum* showing strong inhibition of mycelia growth (Figure 1). However, we do not know whether the inhibition of mycelial growth in *F. graminearum* is directly related to polyamine biosynthesis.

### Changes to the membrane-associated transporting system of the host harboring FgV1-DK21

Along with the reduced amounts of many metabolites such as carbohydrates, gene expression for the transfer of such metabolites or ions from one side of the membrane to the other was greatly suppressed in the host harboring FgV1-DK21. This indicates that virus infection affects the transport of many micro- and macro-elements in host cells at the transcript level. Moreover, this transport system mediates cell-to-cell communication within the host via plasma membranes. For example, transcripts required for cellular communication were highly accumulated at 36 h, suggesting that the virus might first stimulate cell-to-cell communication in fungal host cells, which is necessary to trigger host defense mechanisms against viral pathogens. Indeed, a recent study reported that infection of chlorovirus, *Paramecium bursaria* chlorella virus 1, affects the transport activity of solutes via plasma membranes in *Chlorella*[43]. Plasma membranes are the first barriers to block pathogen attack and can transmit information and molecules between neighboring cells. Viruses utilize plasma membranes to interact with signaling molecules. A previous study suggested that virus infection causes depolarization of the host cell membranes, thus decreasing the transport of solutes by active transporters via plasma membranes [43]. The impairment of plasma membranes by viruses suggests that all materials required for virus replication should be recruited within the host cell [43]. Taken together, these data suggest that FgV1-DK21 might inhibit the transport of diverse metabolites via plasma membranes to the maximize energy required for virus replication within the nucleolus.

### Fungal-specific TFs are key players in the gene expression regulation in *F. graminearum* harboring FgV1-DK21

The expressions of members of the Zn2Cys6 TF family were strongly altered at both the early and late time points. These are known to be fungal-specific, and their functional roles are diverse, including sugar and amino acid metabolism, gluconeogenesis, respiration, vitamin synthesis, cell cycle, chromatin remodeling, nitrogen utilization, peroxisome proliferation, drug resistance, and stress response [30]. The Zn2Cys6 family is the largest TF family in *F. graminearum*, comprising 309 TFs. Of the nine TFs that were differentially expressed at both the early and late time points, six belong to the Zn2Cys6 family, indicating that expression of Zn2Cys6 is necessary in the host harboring FgV1-DK21. Furthermore, the enriched GO terms for nucleic acid binding that were up-regulated at 36 h provide evidence for transcriptional regulation by TFs such as the Zn2Cys6 TF family (Figure 9). To characterize the functional roles of the Zn2Cys6 TF family associated with FgV1-DK21, it is necessary to examine the phenotypes of knock-out mutants. Therefore, the recently generated *F. graminearum* deletion mutant lines will be very useful resources for characterizing the functions of host TFs associated with hypovirulence.

### FgV1-DK21 triggers cAMP-mediated signal transduction in host cells

Fungi utilize cAMP-mediated signal transduction pathways to recognize and respond to diverse environmental stimuli. cAMP signaling is implicated in the regulation of hyphal growth, mating, and gluconeogenesis in many fungi [44,45]. In addition, cAMP and the G-protein alpha subunit coordinate their activities to regulate differentiation and virulence in some fungi. We found that transcripts for genes associated with responses to cAMP were highly accumulated in the late stage of virus infection. This result is consistent with previous data that suggested up-regulation of cAMP levels in the fungal transcriptome by hypovirus infection [46]. Thus, we hypothesize that FgV1-DK21 attenuates the pathogenicity of *F. graminearum* via cAMP-mediated signaling and that this process occurs relatively late after virus infection.

## Conclusions

Recent years have seen extraordinary developments in genome-wide experimental methods. Of these, microarray analyses facilitate an understanding of the dynamic gene expression patterns of target organisms during environmental stimuli such as biotic and abiotic stresses. Here, given the benefits of the available whole genome sequences of *F. graminearum*, we generated a 3′ tiling microarray system covering whole genes. To decipher global transcriptional reprogramming in *F. graminearum* harboring FgV1-DK21 in detail, samples were harvested at two different time points, thus providing lists of differentially expressed genes early and late in the host containing FgV1-DK21 as compared to an uninfected strain. Numbers of differentially expressed genes at the early and late time points were comparable, but the gene lists differed, suggesting time-dependent transcriptional changes. Genes that were up-regulated at the early time point included those involved in protein synthesis, such as ribosome assembly, as well as nucleolus and ribosomal RNA-processing genes, suggesting that FgV1-DK21 strongly modulates translational machinery in *F. graminearum* to maximize viral replication. Moreover, the accumulation of transcripts associated with transcription, such as TFs, indicated that the transcriptional machinery, which is largely regulated by fungal-specific TFs, might be one of the main targets for virus infection. In contrast, genes involved in various metabolic pathways, particularly those that produce carboxylic acids, aromatic amino acids, nitrogen compounds, and polyamines, were highly down-regulated at the early time point (36 h). Interestingly, such components are closely associated with the host defense mechanism. These results suggest that FgV1-DK21 suppresses the production of such defense-related components until the transcriptional and translational machinery in host cells have adjusted to FgV1-DK21 replication. In addition, transport systems associated with membranes were severely damaged by hindering the recruitment of materials for viral replication within host cells. When faced with viral infection, *F. graminearum* tries to establish a defense mechanism by consistently up-regulating genes associated with defense and virulence at the late time point (120 h). Taken together, our data provide strong genome-wide transcriptional evidence of how FgV1-DK21 regulates the transcriptional reprogramming in *F. graminearum*.

## Methods

### Fungal strains and growth conditions

Virus-free and FgV1-DK21-infected *F. graminearum* strain-DK21 were stored in 15% (v/v) glycerol at −80°C and reactivated on PDA at 25°C with a 12 h light–dark cycle. *F. graminearum* cultures used for RNA extractions were grown as described previously [9]. Freshly grown mycelia from PDA media plates were inoculated in 50 or 200 ml complete media (CM) broth [6] and incubated at 25°C for 36 h or 120 h in an orbital shaker (150 rpm). Hyphae were collected by filtering through 3MM paper, washed with distilled water, dried by blotting with paper towels, and frozen at −80°C.

### Trichothecene analysis

Conidia of virus-free and FgV1-DK21-infected *F. graminearum* strains were harvested in 50 ml of CMC broth at 4 days after inoculation. Conidial suspensions (2 × 105 conidia per dish) were grown in 20 ml of defined media containing 5 mM of agmatine [47] in 90 × 15 mm Petri dishes for 7 days prior to filtrate harvests. Three replicates were used for this treatment. Mycotoxin was extracted from the isolates and analyzed with a Shimadzu QP-5000 gas chromatograph–mass spectrometer, as described previously [48]. The trichothecenes were measured based on biomass produced by each strain.

### Preparation of total RNA samples and cDNA synthesis for RT-PCR

Frozen mycelia were pulverized with a mortar and pestle using liquid nitrogen for nucleic acid extraction. Total RNAs were extracted by Iso-RNA Lysis reagent (5 PRIME, Germany). Extracted total RNA was treated with DNase I (Takara, Japan) to remove genomic DNA according to the instructions provided by the manufacturer. These total RNA samples were precipitated with ethanol and resuspended in DEPC-treated water. Next, 5 μg total RNA of each sample was used to synthesize first-strand cDNA with an oligo (dT)_18_ primer and M-MLV reverse transcriptase (Promega, USA) according to the manufacturer’s protocols. All synthesized cDNAs were diluted 1:10 with nuclease-free water for RT-PCR.

### Probe design

Expression profiling was conducted with the *Gibberella zeae* 135k Microarray, which was designed based on the *F. graminearum* sequences released in March 2007 (http://www.broad.mit.edu/annotation/genome/fusarium_group/). The genome contains a total of 13,382 genes. Ten 60-nucleotide-long probes were designed from each gene starting 250 base pairs (bp) ahead of the end of the stop codon and by shifting 10 bp. Thus, these 10 probes covered 150 bp in the 3′ region of the target gene. Mitochondrial genes (50 genes) and selected markers such as *gfp*, *gus*, *hyg*, *bar*, and *kan* were included. In total, 133,612 probes were designed. The average probe size was 60 nucleotides long, and the Tm values were adjusted from 75 to 85°C. The microarray was manufactured by NimbleGen Inc. (http://www.nimblegen.com/). Random GC probes (40,000) to monitor the hybridization efficiency and four corner fiducial controls (225) to assist with overlaying the grid on the image were included.

### cDNA synthesis and microarray hybridization

To assess the reproducibility of the microarray analysis, we repeated the experiment three times with independently prepared total RNAs. Thus, a total of 12 samples were subjected to total RNA isolation and used for microarray analyses. For the synthesis of double-stranded cDNAs, the RevertAidTM H Minus First Strand cDNA Synthesis Kit (Fermentas, Lithuania) was used. In brief, 1 μl oligo dT primer (100 μM) and 10 μl (10 μg) total RNAs were combined and denatured at 70°C for 5 min and renatured by cooling the mixture in ice. First-strand DNA was synthesized by adding 4 μl 5 X First Strand Buffer, 1 μl RiboLockTM Ribonuclease Inhibitor, 2 μl 10 mM dNTP mix, and 1 μl RevertAidTM H Minus M-MuLV Reverse Transcriptase enzyme and incubating at 42°C for 1 h. The reaction was stopped by heating at 70°C for 10 min. To synthesize the second strand, 66.7 μl nuclease free water, 5 μl 10 X reaction buffer for DNA Polymerase I (Fermentas, Lithuania), 5 μl 10 X T4 DNA ligase buffer (Takara, Japan), 3 μl 10 unit/ul DNA Polymerase I (Fermentas, Lithuania), 0.2 μl 5 unit/μl Ribonuclease H (Fermentas, Lithuania), and 0.1 μl 350 unit/μl T4 DNA ligase (Takara, Japan) were added to the first-strand reaction mixture and the reaction was performed at 15°C for 2 h. The double-stranded cDNA mixture was purified using the MinElute Reaction Cleanup Kit (QIAGEN, USA). For the synthesis of Cy3-labeled target DNA fragments, 1 μg double-stranded cDNA was mixed with 30 μl (1 optical density) Cy3-9mer primer (Sigma-Aldrich, USA) and denatured by heating at 98°C for 10 min. The reaction was further proceeded by adding 10 μl 50 X dNTP mix (10 mM each), 8 μl deionized water, and 2 μl Klenow fragment (50 unit/μl, Takara, Japan) and incubating at 37°C for 2 h. DNA was precipitated by centrifugation at 12,000 x *g* after adding 11.5 μl 5 M NaCl and 110 μl isopropanol. Precipitated samples were rehydrated with 13 μl water. The concentration of each sample was determined using a spectrophotometer. A 10 μg aliqout of DNA was used for microarray hybridization. The sample was mixed with 19.5 μl 2 X hybridization buffer (Nimblegen, USA) and finalized to 39 μl with deionized water. Hybridization was performed with an MAUI chamber (Biomicro, USA) at 42°C for 16–18 h. After hybridization, the microarray was removed from the MAUI Hybridization Station and immediately immersed in a shallow 250 ml Wash I (Nimblegen, USA) at 42°C for 10–15 s with gentle agitation, then transferred to a second dish of Wash I and incubated for 2 min with gentle agitation. The microarray chip was then transferred to another dish of Wash II and further washed in Wash III for 1 min with agitation. The slide was dried in a centrifuge for 1 min at 500 g and scanned using a GenePix scanner 4000B (Axon, USA).

### Data analysis

The hybridized microarray chip was scanned with Genepix 4000 B (Axon Instruments) preset with a 5 μm resolution for the Cy3 signal. Signals were digitized and analyzed by Nimblescan (Nimblegen, USA). The grid was aligned to the image with a chip design file called the NDF file. The alignment was assessed by ensuring that the grid’s corners were overlaid on the image corners. This was further checked by uniformity scores in the program. The analysis was performed in a two-part process. First, pair-report files were generated in which the sequence, probe, and signal intensity information for the Cy3 channel were collected. Data-based background subtraction using a local background estimator was performed to improve fold-change estimates on arrays with high background signals. The data were normalized and processed with a cubic spline normalization using quantiles to adjust for signal variation between chips [49]. A probe-level summarization by Robust Multi-Chip Analysis (RMA) using a median polish algorithm implemented in NimbleScan was used to produce call files in order to improve the sensitivity and reproducibility of microarray results [50].

The multiple correction analysis was performed using the limma package in an R computing environment [51]. Linear models implemented in lmFit and empirical Bayes methods implemented in eBayes were applied to assess the differential expression of genes. Genes for which the adjusted P-value or false discovery rate was below 0.05 were collected and further selected. Hierarchical clustering was performed by Acuity 3.1 (Axon Instruments) with similarity metrics based on squared Euclidean correlation, and average linkage clustering was used to calculate the distance of genes. The microarray data were deposited in the NCBI Gene Expression Omnibus (GEO) database with the Accession Number GSE30545.

### GO enrichment analysis

To gain insight into the functions of the differentially expressed genes, GO enrichment analysis was conducted with GOMINER [52,53]. The 8,338 genes were matched to the *M. grisea* sequencing assembly SC5 (http://www.broad.mit.edu/annotation/genome/magnaporthe_grisea/) with scores of 100 and up by BlastP analysis and were used as the total gene set for GO enrichment analysis. The GOMINER program first categorizes each gene according to its GO terms and the mode of gene expression (either down- or up-regulation). Modes of expressions are denoted as under, over, and change. The program then calculates P-values using a one-sided Fisher exact test for the number of categorized GO terms out of the total number of terms. False discovery rate (FDR) values were obtained from 100 randomizations. GO terms for which the FDR was less than 0.05 were selected.

### qRT-PCR analysis

qRT-PCR was performed on Bio-Rad’s CFX96™ Real-time PCR system using gene-specific internal primers. Each reaction mix (10 μl) consisted of 25 ng total cDNA, 5 μl 2 X SsoFast™ EvaGreen® Supermix (Bio-Rad, USA), and 10 pmoles each primer. The thermal profile was as follows: 3 min at 95°C and 40 cycles of 5 s at 95°C, 5 s at 58°C, and melting curve data obtained by increasing the temperature from 65 to 95°C. Elongation factor 1α (EF-1α; FGSG_08811) and cyclophilin (Cyp1; FGSG_07439) were used as internal reference genes to normalize mRNA levels between samples (EF-1α; GenBank accession No. XM388987, Cyp1; GenBank accession No. XM387615). Data were analyzed using the Bio-Rad CFX Manager V1.6.541.1028 software (Bio-Rad, USA). RNA was extracted from three independent replicate experiments, and each PCR product was evaluated in at least three independent experiments, including three technical replicates [54].

## Abbreviations

h: Hours post inoculation; FgV1-DK21: Fusarium graminearum virus 1 strain DK21; TF: Transcription factor; GO: Gene ontology.

## Competing interests

The authors declare that they have no competing interests.

## Authors’ contributions

JY, KML, MS, YWL, and KK designed the experiment. JY, KML, and MS performed cultivation of *F. graminearum* and infection with FgV1-DK21. JY and KM conducted mycotoxin analysis, isolated total RNAs, and conducted RT-PCR. WKC, JY, and KK analyzed the microarray data and interpreted the results. JY and KK coordinated the study. WKC, JY, YWL, and KK wrote the manuscript. All authors read and approved the final manuscript.

## Supplementary Material

Additional file 1 : Table S1.Differentially expressed *F. graminearum* genes at 36 h. Click here for file

Additional file 2 : Table S2.Differentially expressed *F. graminearum* genes at 120 h. Click here for file

Additional file 3 : Table S3.The 20 representative *F. graminearum* genes selected for qRT-PCR validation. Click here for file

Additional file 4 : Table S4.Oligonucleotide primers used for qRT-PCR. Click here for file

Additional file 5 : Table S5.Enriched GO terms of differentially expressed genes. Click here for file

Additional file 6 *: Table S6.F. graminearum* transcription factors which were differentially expressed by FgV1-DK21 infection. Click here for file
